# SERS- and SEIRA-Based Characterization and Sensing of Highly Selective Bradykinin B2 Receptor Antagonists

**DOI:** 10.3390/ijms26168089

**Published:** 2025-08-21

**Authors:** Edyta Proniewicz, Adam Prahl

**Affiliations:** 1 Faculty of Foundry Engineering, AGH University of Krakow, 30-059 Krakow, Poland; 2 Faculty of Chemistry, University of Gdansk, Wita Stwosza 63, 80-308 Gdansk, Poland; adam.prahl@ug.edu.pl

**Keywords:** bradykinin, BK, receptor antagonist, surface-enhanced Raman spectroscopy, SERS, surface-enhanced infrared spectroscopy, SEIRA

## Abstract

One of the major challenges in diagnosing various diseases, including neurological and neurodegenerative disorders, as well as carcinogenesis, is detecting unlabeled neurotransmitters. Surface-enhanced Raman spectroscopy (SERS) and surface-enhanced infrared spectroscopy (SEIRA) are promising methods for neurotransmitter biosensing and bioimaging. These methods are unique in that they are non-destructive and can identify molecular fingerprints. In this study, these methods were used to detect the following potent bradykinin (BK) antagonists: [D-Arg^0^,Hyp^3^,Thi^5^,D-Tic^7^,Oic^8^]BK, [D-Arg^0^,Hyp^3^,Thi^5^,D-Phe^7^,Thi^8^]BK, [D-Arg^0^,Hyp^3^,Igl^5^,D-Phe(5F)^7^,Oic^8^]BK, and [D-Arg^0^,Hyp^3^,Igl^5^,D-Igl^7^,Oic^8^]BK. The peptides were immobilized on a sensor surface consisting of silver (AgNPs) and gold (AuNPs) nanoparticles. These sensors have uniform particle sizes and small size distributions. Thanks to fast synthesis, easy handling, and reproducible results, these sensors enable routine testing. The vibrational structure of these peptides could not be determined using classical vibrational methods (Raman and IR) or surface-enhanced methods (SERS and SEIRA). This work presents the results of that research. Additionally, the SEIRA spectrum for BK or its analogs has not yet been published. This study presents research using SERS and SEIRA that shows that AgNP and AuNP sensors can detect the peptides under investigation. SERS is a more selective method than SEIRA because it allows for the differentiation of peptides based on the enhancement of certain bands in the SERS spectra. Furthermore, each peptide uniquely interacts with AuNPs, whereas all peptides bind to AgNPs via the *C*-terminus in different orientations. Consequently, the AuNP sensor is more selective than the AgNP sensor. Some bands were selected as markers for the sensing of specific peptides.

## 1. Introduction

In 2022, there were 20 million new cancer cases worldwide. This number is expected to increase to 35 million cases per year over the next two decades [1]. The most commonly diagnosed cancers were lung, breast, and colorectal cancers. Most of these cancers are caused by genetic abnormalities in the transformed cells, which can result from exposure to substances that interfere with normal body processes, known as carcinogens. Other cancer-promoting genetic abnormalities may be related to mutations in DNA or damage to mechanisms that control cell growth at the epigenetic and molecular levels. These irregularities may also be present in all cells from birth [2]. The variation between individual cancers is usually influenced by complex interactions between carcinogens and the host genome. One such agent is neurotransmitters. These signaling molecules control the activity of organs and tissues and influence the immune system. Neurotransmitters also promote cell proliferation, progression, and metastasis, thereby a playing role in cancer development [3,4,5]. Cancer tissue secretes endogenous neurotransmitters in response to various environmental and nervous system stimuli [6,7]. Conversely, many malignant tumors overexpress certain types of metabotropic seven-transmembrane receptors belonging to the G-protein-coupled superfamily (GPCR) on their surface. These receptors are found in small cell lung cancer (SCLC) (85–100%), glioblastoma (85%), gastric cancer, pancreatic cancer (75%), prostate cancer (62–100%), breast cancer (62–91%), squamous cell carcinoma (100%), and neuroblastoma (72%). They bind to neurotransmitters secreted by the autonomic nervous system in the brain, the peripheral nervous system, the ganglia, and the adrenal medulla [3,8,9,10,11,12,13,14,15]. Therefore, these receptors can serve as potential cancer markers for imaging and therapy with synthetic antagonists and agonists. These agents can be used for the early diagnosis and detection of lesions expressing these receptors [16,17,18].

Bradykinin (BK), whose primary sequence is shown in Table 1, is a neurotransmitter found in the human body and a natural ligand for type B2 G-protein-coupled receptors. BK mediates the following processes via these receptors: sustained smooth muscle contraction, vascular permeability, mucosal secretion, the stimulation of sensory neurons, the alteration of epithelial cell ion secretion, the release of cytokine from leukocytes, and the production of eicosanoids from various cell types [19]. Notably, BK is considered a growth factor involved in carcinogenesis and neoplastic cell progression [20,21]. Therefore, several studies have focused on developing BK analogs with a significantly higher affinity for B2 receptors and antagonistic or weaker agonistic properties [22,23]. These properties enable BK analogs to effectively compete with the native peptide by preferentially binding to B2 receptors and blocking the signaling pathway, thereby inhibiting neoplastic cell proliferation. BK antagonists also exhibit desirable characteristics for combination therapy. A single analog can selectively stimulate apoptosis in tumors, while inhibiting angiogenesis and matrix metalloproteinase (MMP) activity in treated lung and prostate tumors in nude mice [24].

An example of a highly potent and specific bradykinin B2 receptor antagonist with a nanomolar affinity is [D-Arg-Hyp^3^,Thi^5^,D-Tic^7^,Oic^8^]BK. See Table 1 for the primary sequence. This analog differs from other known BK antagonists because residues 7 and 8 are replaced with more rigid, lipophilic amino acids. It is highly stable and slowly degenerates in human plasma [25]. It prevents pancreatic edema, resulting in hypotension and hemoconcentration. It also facilitates the removal of activated enzymes from the tissue during cerulenin-induced acute pancreatitis [26]. In clinical studies, its efficacy in treating hereditary angioedema and ascites associated with liver cirrhosis has been tested [27]. Another important B2 receptor antagonist is [D-Arg^0^,Hyp^3^,Thi^5^,D-Phe^7^,Thi^8^]BK, which causes vasodilation and can therefore be used to treat hypertension. It induces and influences inflammatory reactions. Consequently, it could be beneficial in treating diseases associated with excessive or chronic inflammation. It is also involved in pain mechanisms and could therefore act as an algogenic substance or as a regulator of pain perception [28]. Understanding this antagonist’s role in pain mechanisms could lead to new analgesics or methods for treating chronic pain [29]. Conversely, D-Arg^0^,Hyp^3^,Igl^5^,D-Igl^7^,Oic^8^]BK reduces the number of leukocytes that adhere to the endothelial surface and improves cerebral microcirculation in gerbils following global cerebral ischemia [30]. An interesting modification of this peptide involves replacing the amino acid at position 8 with L-octahydroindole-2-carboxylic acid (Oic). Recently, several potential inhibitors of protein S binding to ACE2 have been developed based on this non-natural amino acid. These inhibitors prevent SARS-CoV-2 from entering cells, thereby weakening the progression of the 2019 novel coronavirus disease (COVID-19) [31]. Including D-pentafluorophenylalanine at position 7 (Phe(5F)^7^) modifies the peptide structure, improving the potency and selectivity of the [D-Arg^0^,Hyp^3^,Igl^5^,D-Phe(5F)^7^]BK analog toward the B2 receptor while maintaining its antibacterial properties [32,33].

The presence of neurotransmitters, as well as imbalances in their levels, which exhibit dose-dependent effects, may indicate disorders affecting brain function, blood pressure regulation, smooth muscle contraction, inflammatory processes, and cancer growth. Therefore, the ability to detect neurotransmitters is becoming increasingly important in various fields, including the diagnosis of brain and neurological diseases and tumors. This is the motivation for the research in the present study. This study employed spectroscopic methods, including surface-enhanced Raman spectroscopy (SERS) and surface-enhanced infrared spectroscopy (SEIRA), to detect four potent bradykinin antagonists: [D-Arg^0^,Hyp^3^, Thi^5^,D-Tic^7^,Oic^8^]BK, [D-Arg^0^,Hyp^3^,Thi^5^,D-Phe^7^,Thi^8^]BK, [D-Arg^0^,Hyp^3^,Igl^5^,D-Phe(5F)^7^,Oic^8^]BK, and [D-Arg^0^,Hyp^3^,Igl^5^,D-Igl^7^,Oic^8^]BK. The molecular structures of these peptides remain unknown. Another aim of this work is to characterize these antagonists’ structures using vibrational spectroscopy, by means of Raman spectroscopy and attenuated total reflection Fourier transform infrared (ATR-FTIR) spectroscopy.

SERS and SEIRA are highly effective analytical tools for screening and sensing peptides, even at the single-molecule level, and offer exceptional specificity [34,35,36]. In these methods, the analyte is adsorbed onto or placed near a nanometer-sized metal surface, where the metal surface’s plasmon filed is effectively amplified [37]. Silver surfaces have been used in 90% of SERS studies because they most effectively amplify the metal surface plasmon field [38]. Gold has also been used as an SERS-active substrate for its stability and ability to provide surface plasmon resonance conditions in the visible and near-infrared spectral regions. However, gold does not produce as strong an enhancement as silver. In protein screening, selecting a suitable substrate preparation method is crucial because it facilitates routine testing. Therefore, the method should be fast, simple, and inexpensive. It should also allow for the preparation of repeatable, uniform nanometal surfaces and reproducible results. Thus, this study employes colloidal silver (AgNPs) and gold nanoparticles (AuNPs).

Previously the surface- (SERS) and tip-enhanced Raman scattering (TERS) techniques were used to determine the adsorption of bradykinin (BK) and its two isotopically labeled analogs, [(Phe-D_5_)^5^]BK and [(Phe-D_5_)^8^]BK, under various environmental conditions, including peptide concentrations (10^−5^–10^−7^ M) and excitation wavelengths (514.5 and 785.0 nm), the pH of the solution (from pH = 3 to pH = 11), and the roughness and size of the metal surface. Examples include colloidal, suspended Ag nanospheres with a diameter of 10 nm and Ag, Au, and Cu electrode surfaces under different applied electrode potentials (−1.200 V to 0.200 V) in aqueous solutions [39,40,41]. The SERS results indicated that a peptide concentration of 10^−5^ M was optimal for monolayer colloidal coverage. The Phe^5/8^ and Arg^9^ residues of BK generally interacted with the colloidal Ag surfaces. The amide group arranged itself similarly on the Ag surface within the pH range of 3 to 11. At an acidic pH of the solution (pH = 3 to 5), the BK –COO^−^ terminal group bound to the Ag surface as a bidentate (at pH = 3) or monodentate (at pH = 5) chelating ligand. At a pH = 11, the imino group of Arg^9^ was not involved in interacting with AgNPs, likely due to its protonated state (–C = N^⊕^H_2_). Reducing the alkalinity of the solution (pH = 9) caused the deprotonation of the –C = N^⊕^H_2_ group, followed by rearrangement of the group to favor interactions between the lone electron pair on N and Ag. The TERS studies confirmed BK’s behavior on colloidal Ag surfaces at a pH = 7, as proposed based on SERS.

The behavior of four potent B_2_ bradykinin receptor antagonists—including [D-Arg^0^,Hyp^3^,Thi^5,8^,L-Pip^7^]BK, Aaa[D-Arg^0^,Hyp^3^,Thi^5,8^,L-Pip^7^]BK, [D-Arg^0^,Hyp^3^,Thi^5^,D-Phe^7^, L-Pip^8^]BK, and Aaa[D-Arg^0^,Hyp^3^,Thi^5^,D-Phe^7^,L-Pip^8^]BK, adsorbed onto different surfaces, such as colloidal Ag spheres with diameters of approximately 20–25 nm and colloidal Ag nanowires with a diameter ranging from 350 to 500 nm and a length ranging from 2 to 50 µm, as well as an electrochemically roughened Ag surface with a 50–150 nm Ag island on its surface—was also monitored by SERS [42,43,44]. The adsorption of the other five BK analogs, which act as antagonists of the B1 receptor in the presence of bradykinin in the T-REx 293/B1R cell line, was discussed in the context of enhancing the vibrations of the molecular fragments responsible for the biological properties of these analogs at the liquid/AuNPs interface (with a diameter of 20 nm) [45].

## 2. Results and Discussion

**Figure 1:**
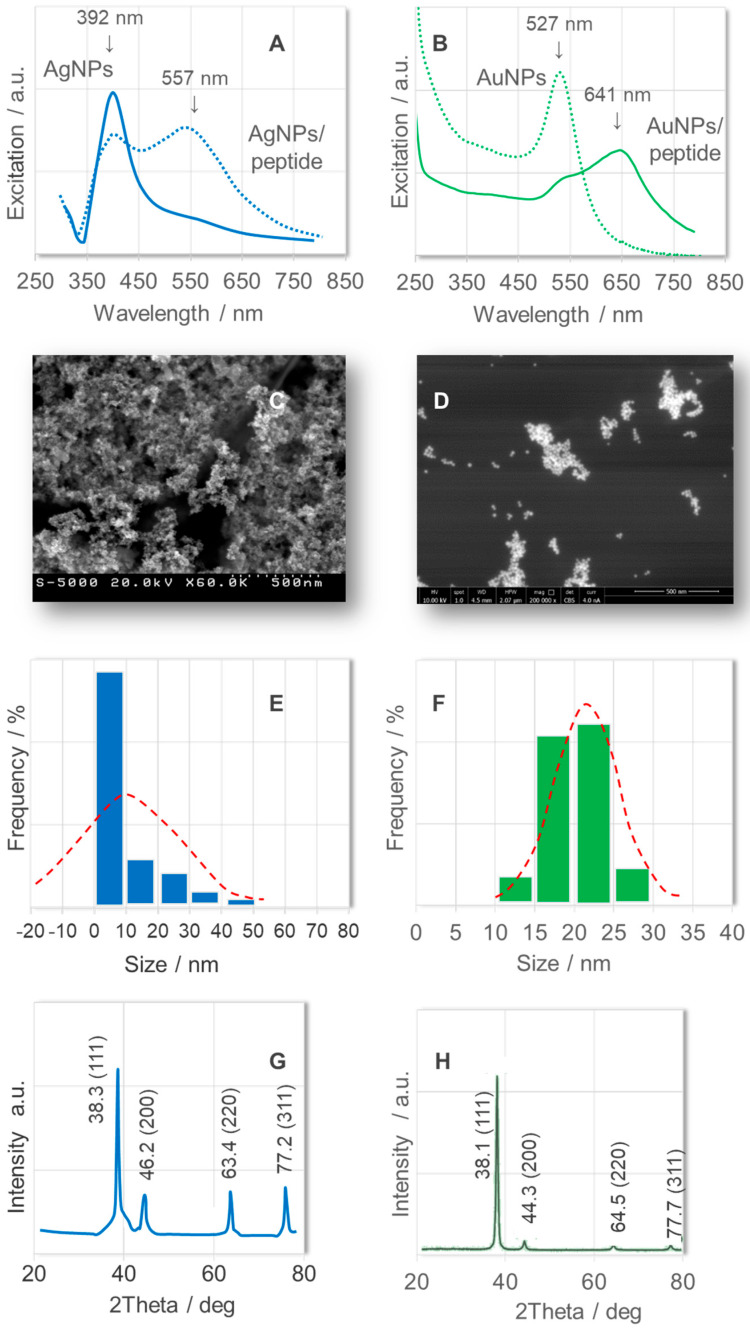
Excitation (UV—Vis) spectra of (**A**)—the aqueous Ag sol (nanoparticles with a diameter of ~10 nm; solid line) and the peptide/AgNPs (dotted lines) and (**B**)—the aqueous Au sol (nanoparticles with a diameter of ~20 nm; solid line) and the peptide/AuNPs (dotted lines). Insets: (**C**)—SEM image of AgNPs (10.0 kV, ×200,000, scale 500 nm); (**D**)—SEM image of AuNPs (10.0 kV, ×200,000, scale 500 nm), (**E**)—DLS analysis of AgNPs; (**F**)—DLS analysis of AuNPs; (**G**)—XRD pattern of AgNPs; and (**H**)—XRD pattern of AuNPs.

Before using Ag and Au nanoparticle sensors to detect peptides, the dimensions and size distribution of these nanoparticles were determined using a UV-Vis spectrophotometer, a scanning electron microscope (SEM), and dynamic light scattering (DLS). Figure 1 shows the excitation spectra of an aqueous solution of AgNPs (Figure 1A, solid line) and AuNPs (Figure 1B, solid line). The plasmon resonance at 392 and 527 nm is due to the collective oscillation of the free electrons in the conduction band of the nanoparticles. This resonance band is accompanied by a full width at half maximum of 80 nm and 50 nm, respectively. These observations suggest the precise control of the particle size, with values of 10 nm and 20 nm, respectively. The SEM images (Figure 1C,D) and DLS analysis (Figure 1E,F) corroborate the UV-Vis results. Additionally, the calculated crystallite size based on XRD patterns (Figure 1G,H) is approximately 10 and 20 nm for AgNPs and AuNPs, respectively. The SEM images (Figure 1C,D) also show aggregated spherical nanoparticles. Nanoparticle aggregates form due to attractive forces, such as van der Waals forces and electrostatic interactions, as well as through the Oswald ripening process and upon molecule adsorption [46,47]. This phenomenon is reflected in the absorption band shift to 557 nm and 641 nm for AgNPs and AuNPs, respectively (see dotted lines in Figure 1A,B).

The AgNPs and AuNPs, with diameters of 10 and 20 nm, respectively, were selected as sensors. This selection was based on previous research involving other neurotransmitters immobilized on nanoparticles of these sizes [39,43,45,48,49]. This allows us to compare the present results with studies on different neurotransmitters under similar experimental conditions. This is important when sensing different neurotransmitters.

**Figure 2:**
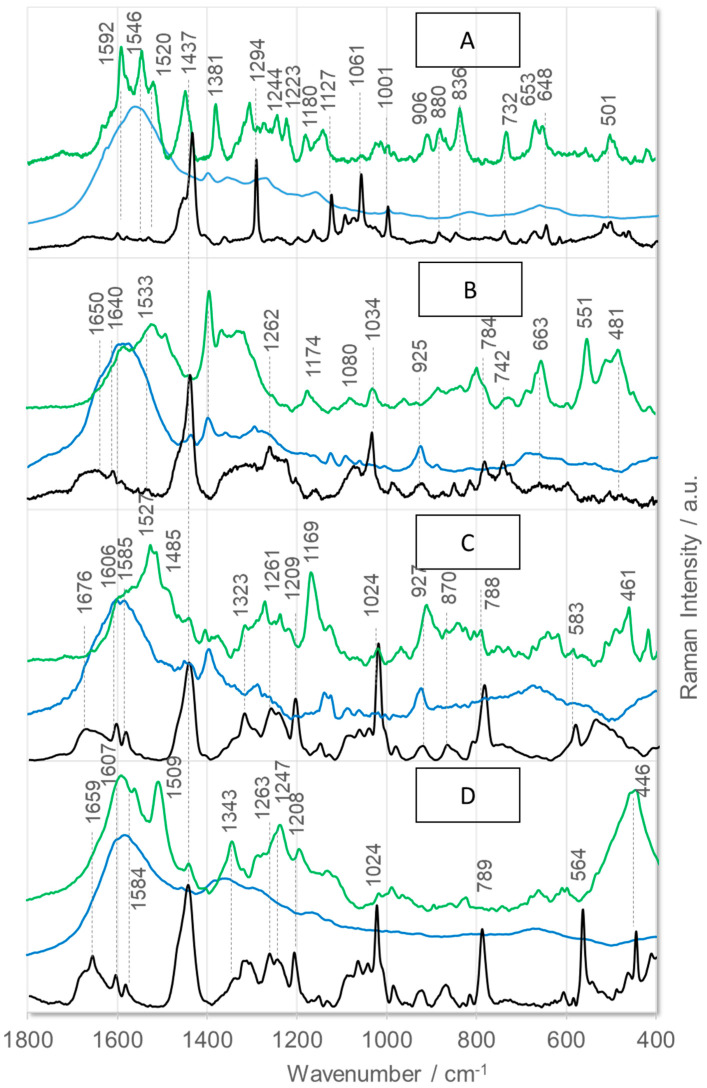
Raman (black) and SERS spectra of (**A**)—[D—Arg^0^,Hyp^3^,Thi^5^,D—Phe^7^,Thi^8^]BK, (**B**)—[D—Arg^0^,Hyp^3^,Thi^5^,D—Tic^7^,Oic^8^]BK, (**C**)—[D—Arg^0^,Hyp^3^,Igl^5^,D-Phe(5F)^7^,Oic^8^]BK, and (**D**)—[D—Arg^0^,Hyp^3^,Igl^5^,D—Igl^7^,Oic^8^]BK immobilized on AgNPs (blue line) and AuNPs (green line).

**Figure 3:**
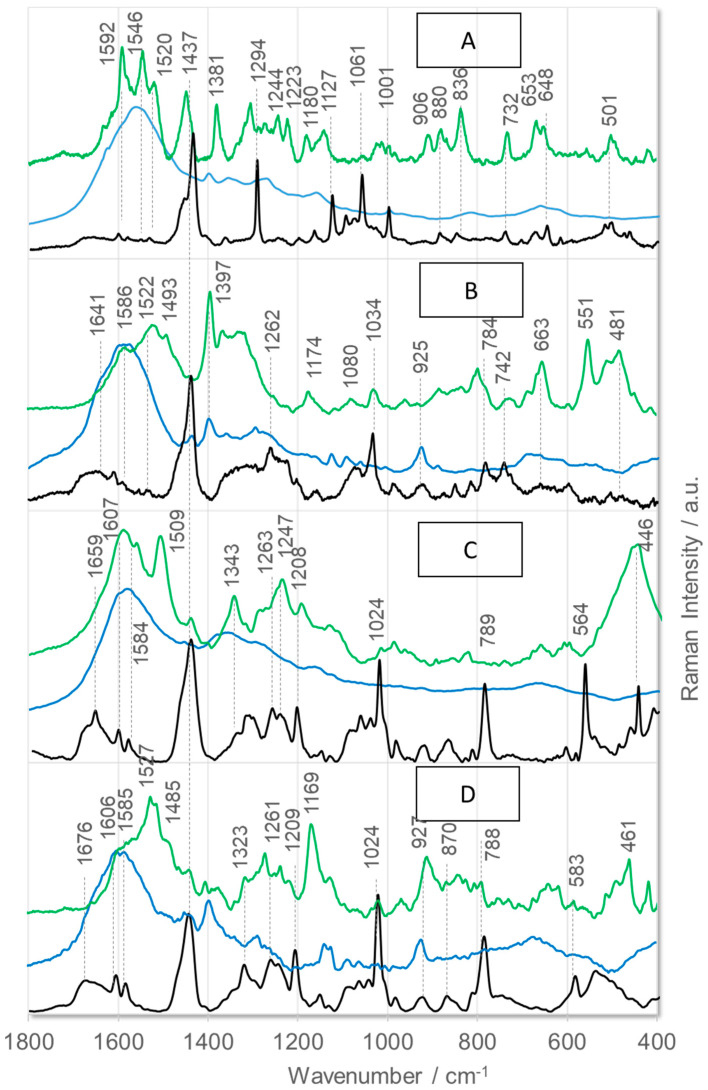
ATR—FTIR (black) and SEIRA spectra of (**A**)—[D—Arg^0^,Hyp^3^,Thi^5^,D—Phe^7^,Thi^8^]BK, (**B**)—[D—Arg^0^,Hyp^3^,Thi^5^,D—Tic^7^,Oic^8^]BK, (**C**)—[D—Arg^0^,Hyp^3^,Igl^5^,D—Phe(5F)^7^,Oic^8^]BK, and (**D**)—[D—Arg^0^,Hyp^3^,Igl^5^,D—Igl^7^,Oic^8^]BK immobilized on AgNPs (blue line) and AuNPs (green line).

Figure 2 shows the SERS spectra of the four antagonists detected on AgNP (blue lines) and AuNP (green lines) surfaces. To facilitate the accurate interpretation of the SERS results, the corresponding Raman spectra (black lines) are shown for comparison. Figure 3 shows the corresponding SEIRA spectra on AgNPs (blue lines) and AuNPs (green lines), together with the ATR-FTIR spectra (black lines). It is important to note that the vibrational structure of these compounds has not yet been determined. Therefore, Table 2 and Table 3 summarize the proposed assignments of the observed bands in the presented spectra to the vibrations of the corresponding peptide fragments. However, the following discussion will focus exclusively on the bands that effectively recognize/sense these antagonists.

Before analyzing the results of immobilized neurotransmitters on sensing surfaces, four aspects must be considered. The first concerns the primary structure of neurotransmitters and their differences. This is important for describing the molecules’ geometry on metal surfaces because surface-enhanced methods only enhance molecule fragments on or close to the sensor surface. Peptide fragments attach to the sensor surface based on their chemical structure. Second, the bands that appear in the vibrational spectra must be assigned to the corresponding normal modes of the peptide fragments. Third, surface plasmon resonance refers to the oscillation of a metal’s surface charge density. This oscillation can be amplified by electromagnetic radiation at frequencies close to the metal’s surface plasmon frequency. The physics of the processes underlying Raman and infrared absorption spectroscopy determine the selectivity for detecting specific peptide fragments. Specifically, in Raman spectroscopy, the transition probability depends on the polarizability of the molecule, whereas in infrared spectroscopy, it depends on the dipole moment. Therefore, only molecules with a non-zero dipole moment produce an IR spectrum. Polar fragments of molecules meet this condition. Consequently, infrared spectroscopy mainly detects polar fragments, while Raman spectroscopy mainly detects nonpolar fragments.

Fourth, changes in the bandwidth, wavenumber, and intensity between the normal and surface-enhanced spectra (e.g., Raman vs. SERS and ATR-FTIR vs. SEIRA) enable the determination of the geometry of molecule fragments located on or near the sensor surface. However, this is only possible for bands that do not overlap with other bands in the spectrum. In brief, the adsorption mechanism of different benzene ring derivatives on roughened metal surfaces via π-electron systems has been widely investigated [50,51,52,53,54]. Surface selection rules have been most thoroughly described for the electromagnetic enhancement (EM) of molecules of a high symmetry immobilized on a metal surface [52,53,55]. According to these rules, vibrations perpendicular to the surface (which draw their intensities from the Raman polarizability component α_zz_) are expected to be more enhanced than parallel vibrations [50,51,56]. This means that, for a perpendicular ring orientation, in-plane vibrations should be more enhanced than out-of-plane ones. Therefore, for a flat ring orientation, A_2_ symmetry vibrations contain a larger polarizability tensor component normal to the surface than B_2_ modes. The opposite is true for vertical adsorption geometry. Additionally, upon π adsorption, benzene ring bands involving C–C stretching vibrations shift to lower wavenumbers due to the anticipated back-donation of the electron density from the metal surface to the π* antibonding orbitals of the benzene ring [57]. Gao and Weaver reported that the ν_12_ and ν_18a_ modes of alkylobenzenes shift down by 10–15 cm^−1^ upon adsorption7. Conversely, the observed downshift of 4–5 cm^−1^ for the corresponding modes of halogenobenzenes was interpreted as evidence of the absence of a direct interaction between the π-electron system and the metal surface. Furthermore, a significant benzene ring band broadening was reported in the case of the π-adsorption, caused by the interaction between the benzene ring and the metal surface [57].

As the peptide abbreviations indicate, these peptides have different amino acid sequences at positions 5, 7, and 8. Specifically, peptides [D-Arg^0^,Hyp^3^,Thi^5^,D-Phe^7^,Thi^8^]BK and [D-Arg^0^,Hyp^3^,Thi^5^,D-Tic^7^,Oic^8^]BK contain L-thienylalanine (Thi) at position 5. Thus, the vibrations of this aromatic amino acid’s side chain should be preferentially enhanced in the SERS spectra if it is in proximity to the sensor surface, since aromatic rings have a high affinity for the metal surfaces [58,59,60,61]. Similarly, the vibrations of the phenyl ring should be enhanced in the SERS spectra because this ring is present in the side chains of the amino acids such as D-phenylalanine (D-Phe) and D-1,2,3,4-tetrahydroisoquinoline-3-carboxylic acid (D-Tic) at position 7 in the sequences of peptides [D-Arg^0^,Hyp^3^,Thi^5^,D-Phe^7^,Thi^8^]BK and [D-Arg^0^,Hyp^3^,Thi^5^,D-Tic^7^,Oic^8^]BK, respectively. The phenyl ring also occurs in the side chains of two other non-natural amino acids: L-/D-2-indanylglycine (L-/D-Igl) and D-pentafluorophenylalanine (D-Phe(5F)). The former occurs at positions 5 and 7 in the [D-Arg^0^,Hyp^3^,Igl^5^,D-Igl^7^,Oic^8^]BK sequence. In the sequence of [D-Arg^0^,Hyp^3^,Igl^5^,D-Phe(5F)^7^,Oic^8^]BK, D-Igl and D-Phe(5F) are found at positions 5 and 7, respectively. Additionally, amino acids with side chains containing an atom capable of interacting with the metal surface via a free electron pair primarily enhance signals in SERS/SEIRA spectra. These functional groups include arginine (Arg, –N(H)C(=NH)NH_2_ fragment), L-proline (Pro, >N– fragment), L-hydroxyproline (Hyp, >N– and –OH fragments), L-octahydroindole-2-carboxylic acid (Oic, >N– fragment), D-1,2,3,4-tetrahydroisoquinoline-3-carboxylic acid (Tic, >N– fragment), and L-thienylalanine (Thi, –S– fragment), as well as amide bonds (–C(=O)NH–).

The characteristic modes of these peptide fragments are briefly summarized below:

- Phe: at 1604 cm^−1^ [ν_8a_], 1584 cm^−1^ [ν_8b_], 1204 cm^−1^ [ν_7a_], 1030 cm^−1^ [ν_18a_], 1002 cm^−1^ [ν_12_], and 619 cm^−1^ [ν_6b_] [62];
- Phe(5F): at 1662 cm^−1^ [ν_8a_], 1622 cm^−1^ [ν_8b_], 1552/1514 cm^−1^ [ν(CC)], 1174 cm^−1^ [ν(CF)], 1020 cm^−1^ [ν(CF)], 782 cm^−1^ [ρ_t_(CF)], and 655 cm^−1^ [ρ_t_(CCCC)] [63];
- Thi: at 1409 cm^−1^ [ν(C=CH) + ν(CC)+ρ_r_(CH)], 1363 cm^−1^ [ρ_r_(CH) + ν(C=C)], 1036 cm^−1^ [ν(CC) + ρ_r_(CH)], 986 cm^−1^ [ρ_r_(CH_2_)], 850 and 817 cm^−1^ [ν(CC) + δ(ring)], and 667 cm^−1^ [ρ_w_(CH)] [45];
- Arg: 1574 cm^−1^ [ρ_s_(NH_2_)], 1466–1218 cm^−1^ [different CH_2_ group vibrations], 1176 and 1135 cm^−1^ [ρ_r_(NH_2_)], 1085 and 1068 cm^−1^ [ν(CN)], 997 and 971 cm^−1^ [ρ_r_(CH_2_)], 900–850 cm^−1^ [ν(CC)], 848 cm^−1^ [ρ_w_(NH)], 793 cm^−1^ [ρ_r_(NH)], 746 cm^−1^ [ρ_r_(CH_2_)], and 524 cm^−1^ [ρ_t_(NH_2_)] [64];
- Pro: 1470 cm^−1^ [δ(CH_2_) + imide-II], 1438 cm^−1^ [imide-II + δ(CH_2_)], 1317 cm^−1^ [δ(CC_α_H)], 1286 cm^−1^ [ρ_r/t_(CH_2_)], 1263, 1237, 1178, and 1102 cm^−1^ [δ(CH_2_) and/or δ(NH)], 983 and 950 cm^−1^ [ν(CC) + ν(CCN)], 850 cm^−1^ [ρ_r_(C_β_H_2_], 780 cm^−1^ [ρ_r_(C_γ_H_2_], 530 cm^−1^ [δ(CCN)], and 480 cm^−1^ [pyrrolidine deformations] [65,66];
- Tetrahydroisoquinoline: 1602, 1582, 1460, 1440, 1220, 1196, 1025, 724, 590, 510, and 440 cm^−1^ [67];
- Amide bonds of disordered structures: 1660 m^−1^ [amide-I], 1544 cm^−1^ [amide-II], 1240 cm^−1^ [amide-III], and 644 cm^−1^ [amide-V] [68].

As mentioned earlier, the blue lines in Figure 2 show the SERS spectra of the four BK antagonists on the AgNP surface. These spectra are characterized by a distinct, broad band centered at approximately 1586 cm^−1^ with a full width at half maximum (FWHM) of ~140 cm^−1^. See Table 2 for the exact band wavenumber of each peptide. In this wavenumber range, bands due to vibrations of the amide bond, aromatic ring, carboxyl, and amino groups are expected to be enhanced. Therefore, it can be assumed that the bands resulting from vibrations of these peptide fragments overlap, resulting in a broad, intense band. The statement that these peptide fragments interact with AgNPs is supported by moderately enhanced bands at ~925 cm^−1^ [ν(CC_OO–_)], 1123 cm^−1^ [a-ring ρ_t_(CH_2_), where “a-” stands for an aliphatic ring], 1397 cm^−1^ [ν_s_(COO^−^)], 1438 cm^−1^ [δ(CH_2_) and imide II], and 1457 cm^−1^ [δ(CH_2_) + imide II/Arg ρ_b_(CH_2_)/a-ring ρ_s_(CH_2_)] in the spectra of [D-Arg^0^,Hyp^3^,Igl^5^,D-Igl^7^,Oic^8^]BK (Figure 2D) and [D-Arg^0^, Hyp^3^,Thi^5^,D-Tic^7^,Oic^8^]BK (Figure 2B). In the SERS spectrum of [D-Arg^0^,Hyp^3^,Thi^5^,D-Tic^7^,Oic^8^]BK, the 1438 cm^−1^ SERS signal is more intense than the 1457 cm^−1^ spectral feature. In contrast, the intensities of these two bands are reversed in the [D- Arg^0^,Hyp^3^,Igl^5^,D-Igl^7^, Oic^8^]BK SERS spectrum.

Additionally, the 1139 cm^−1^ band [ρ_r_(NH_2_)]—absent in the [D-Arg^0^,Hyp^3^,Thi^5^,D-Tic^7^,Oic^8^]BK SERS spectrum—is enhanced in the [D-Arg^0^,Hyp^3^,Igl^5^,D-Igl^7^,Oic^8^]BK SERS spectrum on AgNPs. Two other bands in these peptides’ SERS spectra deserve special attention: the ~1270 cm^−1^ band [a-ring ρ_t_(CH_2_)] and the ~1296 cm^−1^ band [a-ring ν(CN) + ρ_b_(CCH) + ρ_r/t_(CH_2_)/imide II]. The enhancement of these bands suggests that both peptides may interact with the AgNP sensor surface through the deprotonated *C*-terminal carboxyl group and the imide bond formed by the Oic^8^ residue. For the [D-Arg^0^,Hyp^3^,D-Igl^7^,Oic^8^]BK peptide, the –NH_2_ group of the arginine side chain interacts with the surface, indicating that the arginine side chain is directed toward the AgNPs.

In the SERS spectra of the other two peptides immobilized on AgNPs, the bands attributable to the vibrations of the carboxylate group are only weakly enhanced (see Figure 2, blue traces A and C). Additionally, the ν_s_(COO^−^) mode appears as a medium-intensity band at 1352 cm^−1^ [aliphatic-ring combination mode] in the [D-Arg^0^,Hyp^3^,Igl^5^,D-Phe(5F)^7^,Oic^8^]BK spectrum (Figure 2C). The SERS signal at 1352 cm^−1^ is also enhanced in the spectrum of [D-Arg^0^,Hyp^3^,Thi^5^,D-Phe^7^,Thi^8^]BK on AgNPs (Figure 2A). However, it is less intense and does not overlap with other bands in this wavenumber range. This behavior can be explained by the fact that the *C*-terminus of these peptides is located at a certain distance from the AgNP surface, with the aliphatic ring facing the surface. This could also indicate that the aliphatic ring of the side chain is on the opposite side of the chain from the carboxyl group. The assignment of the 1352 cm^−1^ band to the aliphatic ring vibrations is supported by the enhancement of other SERS signals of the aliphatic ring at ~1166 cm^−1^ [aliphatic ring ρ_t_(CH_2_)] and ~811 cm^−1^ [aliphatic ring breathing vibrations]. Additionally, the [D-Arg^0^,Hyp^3^,Thi^5^,D-Phe^7^,Thi^8^]BK SERS spectrum on AgNPs shows a weak enhancement of the Phe ν_12_ mode at 996 cm^−1^. This band’s intensity is evidently lower than the corresponding Raman band’s intensity, and its wavenumber is shifted by 5 cm^−1^ to lower wavenumbers. These observations suggest that the phenyl ring is arranged more or less parallel to the AgNP surface [43].

The spectral patterns of peptides immobilized on an AuNP surface differ from those on an AgNP surface. See Figure 2 (green traces) for details. However, there is a certain degree of similarity in the wavenumber range from 1500 to 1600 cm^−1^. This phenomenon occurs due to the overlap of several modes, including those corresponding to the amide bond, aromatic rings, and amine and carboxylate groups.

This results in the formation of a broad envelope of bands with clear maxima. Unlike AgNPs, more discrepancies are observed in the spectra of the individual peptides on AuNPs. In the SERS spectrum of [D-Arg^0^,Hyp^3^,Igl^5^,D-Phe(5F)^7^,Oic^8^]BK on AuNPs (Figure 2C, green trace), the bands at 988, 1195, 1239, 1345, 1442, 1509, 1560, and 1591 cm^−1^, mainly attributed to the Phe(5F) side chain vibrations, are clearly enhanced. In the SERS spectra of [D-Arg^0^,Hyp^3^,Thi^5^,D-Tic^7^,Oic^8^]BK (Figure 2B) and [D-Arg^0^,Hyp^3^,Thi^5^,D-Phe^7^,Thi^8^]BK (Figure 2A) on AuNPs, the ν_s_(COO^−^) mode exhibits an intermediate intensity (see Table 2 for the wavenumbers of the bands). However, this band is absent from the SERS spectrum of [D-Arg^0^,Hyp^3^,Igl^5^,D-Igl^7^,Oic^8^]BK (Figure 2D, green line). In the [D-Arg^0^,Hyp^3^,Thi^5^,D-Tic^7^,Oic^8^]BK spectrum on AuNPs, the ν_s_(COO^−^) band is accompanied by SERS signals at 1583, 1522, 1283, 1174, 959, and 481 cm^−1^, which correspond to the vibrations of the aliphatic ring of the Tic^7^ residue. The [D-Arg^0^,Hyp^3^,Thi^5^,D-Phe^7^,Thi^8^]BK SERS spectrum also shows bands at 1592, 1546, 1520, 982, 836, and 653 cm^−1^. The first three bands overlap, resulting in a pronounced envelope of the bands attributable to amide and phenylalanine ring vibrations. The remaining three spectral features are due to thienylalanine ring vibrations. In contrast, the prominent bands at 1564 and 1485 and 1127 and 461 cm^−1^ in the SERS spectrum of [D-Arg^0^,Hyp^3^,Igl^5^,D-Igl^7^,Oic^8^]BK on AuNPs (Figure 2D, green line) correspond to the amide/imide bond and the aliphatic ring vibrations, respectively.

Figure 3 shows the ATR-FTIR and SEIRA spectra of the investigated BK analogs on AgNPs and AuNPs. The analysis of these spectral profiles reveals that the ATR-FTIR and SEIRA spectra of the individual peptides are similar. This is not surprising, as infrared spectroscopy is important for identifying and characterizing primary chemical functional groups (polar groups), unlike Raman spectroscopy. Briefly, the SEIRA spectra of all peptides on AgNPs are dominated by two modes: amide II at 1580 cm^−1^ and ν_s_(COO^−^) at 1397 cm^−1^. Table 3 shows the specific wavenumbers for each peptide. The 1580 cm^−1^ spectral feature is symmetric, with a full width at half maximum (FWHM) of ~65 cm^−1^ for [D-Arg^0^,Hyp^3^,Thi^5^,D-Phe^7^,Thi^8^]BK (Figure 3A, blue line), [D-Arg^0^,Hyp^3^,Thi^5^, D-Tic^7^,Oic^8^]BK (Figure 3B), and [D-Arg^0^,Hyp^3^,Igl^5^,D-Phe(5F)^7^,Oic^8^]BK (Figure 3C). However, the FWHM is 94 cm^−1^ for the broadened band of [D-Arg^0^,Hyp^3^,Thi^5^,D-Phe^7^,Thi^8^]BK (Figure 3C), which has an asymmetric shape with two shoulders at 1660 cm^−1^ [amide I] and 1533 cm^−1^ [ν_as_(COO^−^)]. The broadening of this band is due to the considerable intensity of the SERS signal at 1660 cm^−1^. This band is also present in the SEIRA spectra of the other three peptides on AgNPs, though it shows only a slight enhancement. The second strong SERS signal is at approximately 1397 cm^−1^ (FWHM = 51 cm^−1^) and exhibits two shoulders at approximately 1447 cm^−1^ [imide II] and ~1368 cm^−1^ [ρ(CH_2_)]. Several other weak bands are enhanced at ~1302 cm^−1^ [ν(CN)], ~1251 cm^−1^ [amide III], 1078 cm^−1^ [ν(CN)], 903 cm^−1^ [ν(CC_OO–_)], 841 cm^−1^ [ν(CC) + δ(NH)], 703 cm^−1^, and 638 cm^−1^ [ρ_b_(COO)/amide V]. However, these bands do not allow the peptides to be distinguished.

The SEIRA spectra of peptides immobilized on the AuNP surface show an intense band in the wavenumber range of 1700–1500 cm^−1^. The analysis of this band’s shape indicates that it results from the overlap of two bands: one at 1660 cm^−1^ [amide I] and one at 1647 cm^−1^ [ν_as_(C=N), δ(NH_2_)]. In each peptide spectrum, the band at the lower wavenumber is more intense than the band at the higher wavenumber. Additionally, four other bands are enhanced in these spectra: one at ~1545 cm^−1^ [amide II], one at ~1455 cm^−1^ [δ(CH_2_) + imide II], one at ~1251 cm^−1^ [amide III], and one at ~1068 cm^−1^ [ν(NC)]). These bands indicate an analogous enhancement in the peptide spectra.

## 3. Materials and Methods

### 3.1. Synthesis of Bradykinin Antagonists

[D-Arg^0^,Hyp^3^,Thi^5^,D-Tic^7^,Oic^8^]BK, [D-Arg^0^,Hyp^3^,Thi^5^,D-Phe^7^,Thi^8^]BK, [D-Arg^0^,Hyp^3^, Igl^5^,D-Phe(5F)^7^,Oic^8^]BK, and [D-Arg^0^,Hyp^3^,Igl^5^,D-Igl^7^,Oic^8^]BK were synthesized via the solid-phase method using the Fmoc strategy and starting from Fmoc-Wang resin (GL Biochem, Shanghai, 1% DVB, 100–200 mesh) [69]. The resin load for the first protected amino acids was 0.40 mmol/g. The Fmoc protection group was removed using 20% piperidine in DMF. The respective Fmoc-amino acids were activated in situ with a threefold excess using HATU (1 equiv.)/HOAt in a DMF/NMP (1:1 *v*/*v*) mixture containing 1% Triton. The coupling reactions were base-catalyzed with NMM. All of the Fmoc-protected amino acids were purchased from NovaBiochem (Bad Soden, Germany). The peptide was cleaved from the resin, and side chain deprotection was performed by treating it with a trifluoroacetic acid (TFA)/water (H_2_O)/trisopropylsilane (TIS) mixture (95.5:2.5:2.5 *v*/*v*/*v*) for four hours. The total volume of the TFA filtrate was reduced to approximately one millimeter by evaporation in vacuo. The peptide was precipitated with a cold diethyl ether and filtered through a Schott funnel. The peptides were purified by semi-preparative high-performance liquid chromatography (HPLC). HPLC was performed on a Waters (analytical and semi-preparative) chromatograph equipped with a UV detector (λ = 226 nm). Peptide purity was determined using a Discovery HS C_18_ column (5 μm, 100 Å; 250 × 4.6 mm). The solvent systems were [A] 0.1% aqueous trifluoroacetic acid (TFA) and [B] 80% acetonitrile in 0.1% aqueous TFA (*v*/*v*). The peptides were separated using a linear gradient of [B] ranging from 1 to 80% over 30 min at a flow rate of 1 mL/min. Semi-preparative HPLC was performed using a Kromasil C_8_ column (5 μm, 100 Å; 16 × 250 mm) with a linear gradient of [B] from 13 to 43% at a flow rate of 8 mL/min. Mass spectra of the peptides were recorded using a Bruker BIFLEX III MALDI TOF mass spectrometer (Bruker, Bremen, Germany) with ionization by a 337 nm nitrogen laser.

### 3.2. Synthesis of Colloidal Silver Nanoparticles (AgNPs)

Silver nitrate (AgNO_3_) and sodium borohydride (NaBH_4_) were obtained from Merck Life Science Sp. z o.o. (Poznań, Poland) and used without further purification. Three batches of an aqueous solution of AgNPs were prepared according to the procedure described by Creighton et al. (reduction of AgNO_3_ by NaBH_4_) [70]. Briefly, 5 mL of a 1·10^−3^ M AgNO_3_ solution was added dropwise to 15 mL of a 2·10^−3^ M NaBH_4_ solution. The solution was kept cool over an ice bath at 4 °C while stirring vigorously. All solutions were prepared with deionized water (18 MΩ cm^−1^). After adding all of the AgNO_3_, the solution was kept in the ice bath for about one hour. As a result, 10 nm AgNPs were obtained in an aqueous solution.

### 3.3. Synthesis of Colloidal Gold Nanoparticles (AuNPs)

Three batches of 20 nm AuNPs, containing 0.01% HAuCl_4_ and stabilized in citrate buffer, were purchased from Merck Life Science Sp. z o.o. (Poznań, Poland). The AuNPs were produced using a modified tannic acid/citrate method.

### 3.4. Sample Preparation for SERS and SEIRA Measurements

Based on previous studies, the optimal concentrations of colloids and neurotransmitters were determined to be those at which well-enhanced, reproducible spectra were measured.

Aqueous peptide solutions were prepared by dissolving each peptide in deionized water (0.08 μS/cm; 10^−4^ M concentration). Then, 10 µL of the peptide solution was mixed with 20 µL of the NPs solution and incubated for 15 min prior to measurements. The incubation time was chosen as 15 min because the spectrum could not be measured immediately after mixing the colloid with the sample solution. Spectra measured at incubation times above 15 min corresponded to spectra measured after 15 min of incubation. Thus, a 15 min incubation time was chosen.

### 3.5. Raman and Surface-Enhanced Raman Spectroscopy (SERS) Measurements

Raman spectra were collected for solid peptides, and SERS spectra were measured for peptides in an aqueous solution.

Both types of spectra were collected using an InVia Raman spectrometer (Renishaw, Wotton-under-Edge, UK), which was equipped with an air-cooled charge-coupled device (CCD) detector and a Leica microscope with a 20× objective. The spectral resolution was set to 4 cm^−1^. The 785 nm line of a continuous-wave diode laser served as the excitation source. The laser power at the laser output was set to 20 mW.

Three batches of each colloid were examined, with SERS spectra recorded from three different drops of the peptide/nanoparticle mixture for each batch (for a total of nine spectra).

The exposure time for each Raman and SERS measurement was typically 40 s with four accumulations (a series of four spectra, each accumulated for 40 s, for a total of 160 s). The spectra obtained from each series were reproducible with slight differences in the intensities of some bands observed (up to 5%). No spectral changes that could be associated with sample decomposition were observed during the measurements.

### 3.6. Attenuated Total Reflection Infrared Spectroscopy (ATR-FTIR) and Surface-Enhanced Infrared Spectroscopy (SEIRA) Measurements

ATR-FTIR spectra were collected for solid peptides, and SEIRA spectra were measured for peptides in an aqueous solution.

Three batches of each colloid were examined, and SERS spectra were recorded from three different peptide/nanoparticle mixtures for each batch (for a total of nine spectra). Briefly, the peptide/nanoparticle mixture was loaded onto a diamond ATR adapter and left to dry. Unbound peptide molecules were removed by washing with deionized water. Then, the adapter was allowed to dry. This process was repeated three times.

Spectra were recorded using a Thermo Scientific Nicolet 6700 FTIR spectrometer (Thermo Fisher Scientific, Waltham, MA, USA) equipped with a diamond ATR accessory. The measurement conditions were a resolution of 4 cm^−1^ and 128 scans. The spectra were recorded three times, each with 128 scans. The spectra obtained from the series were reproducible, with slight differences (up to 5%) in the intensities of some bands observed. No spectral changes that could be associated with sample decomposition were observed during the measurements.

### 3.7. Scanning Electron Microscopy (SEM) Imaging

Ten microliters of an aqueous solution of NPs was deposited onto a silicate plate and dried in a vacuum dryer.

The scanning electron microscope (SEM) images of the AuNPs were obtained using a Versa 3D Dual Beam system (Thermo Fisher Scientific, Waltham, MA, USA) with an energy beam of 10.0 kV.

### 3.8. Dynamic Light Scattering (DLS) Measurements

The volumetric particle size distribution was determined using dynamic light scattering (DLS) measurements with a Zetasizer Nano ZS analyzer (Malvern Instruments, Worcestershire, UK). The analyzer was equipped with an avalanche photodiode with a quantum efficiency of greater than 50% at 633 nm, and it operated at a temperature of 25 °C. Prior to the measurements, a 0.1 wt% SnO_2_ nanoparticle suspension was prepared in distilled water. The suspension was then sonicated for 15 min at 20 W in continuous mode using a Branson SFX250 ultrasonic homogenizer (Emerson, Wallingford, CT, USA).

### 3.9. X-Ray Powder Diffraction (XRD) Pattern Measurements

XRD patterns were obtained using an Aeris diffractometer (PANalytical, Almelo, The Netherlands) with Cu Kα_1_ radiation (λ = 1.5405980 Å). Measurements were taken over an angular range of 10° to 80° 2θ.

## 4. Conclusions

One of the major challenges in diagnosing various diseases, including neurological and neurodegenerative disorders of the central nervous system, as well as carcinogenesis, is label-free neurotransmitter detection. Many effective neurotransmitter detection methods have been developed, including magnetic, microdialytic, electrochemical, optical, and spectroscopic techniques. Among these methods, surface-enhanced Raman scattering (SERS) and surface-enhanced infrared spectroscopy (SEIRA) are promising for biosensing and bioimaging due to their unique non-destructive nature and their ability to identify molecular fingerprints.

In this study, these methods were used to detect four potent bradykinin antagonists in an aqueous solution containing AgNPs and AuNPs. Colloids, which were prepared using methods that allow for the control of the particle size and produce a narrow particle size distribution, were chosen as sensors. Colloids were chosen because they can be synthesized quickly, are easy to handle, and are ideal for routine analyses. The focus of peptide sensing should be on detection rather than sensor fabrication.

The results demonstrate that the AgNP and AuNP sensors can detect the peptides under investigation. Studies with SERS and SEIRA have shown that SERS is a more selective method than SEIRA because it is possible to distinguish peptides based on the enhancement of specific bands in SERS spectra. In the case of AgNPs, the bands at ~1397, 1352, 1136, and 996 cm^−1^ can serve as peptide marker bands—that is, bands that allow for peptide identification. For [D-Arg^0^,Hyp^3^, Thi^5^,D-Phe^7^,Thi^8^]BK, the 996 cm^−1^ band serves as a marker band—it is only enhanced in the SERS spectrum of this peptide on AgNPs. The intense 1397 cm^−1^ band is diagnostic of [D-Arg^0^,Hyp^3^,Thi^5^,D-Tic^7^,Oic^8^]BK, and the 1397 and 1134 cm^−1^ SERS signals are marker bands for [D-Arg^0^,Hyp^3^,Igl^5^,D-Phe(5F)^7^,Oic^8^]BK. The low intensity of the 1395 cm^−1^ band and the enhancement of the 1352 cm^−1^ band are two parameters that can be used to distinguish [D-Arg^0^,Hyp^3^,Igl^5^,D-Phe(5F)^7^,Oic^8^]BK from other peptides. The modes of the pentafluoro-substituted phenyl ring ([D-Arg^0^,Hyp^3^,Igl^5^,D-Phe(5F)^7^,Oic^8^]BK), the amide/imide bond ([D-Arg^0^,Hyp^3^,Igl^5^,D-Igl^7^, Oic^8^]BK), the thienylalanine ring and the –COO^−^ group ([D-Arg^0^,Hyp^3^,Thi^5^,D-Phe^7^,Thi^8^]BK), and the Tic ring and the –COO^−^ group ([D-Arg^0^,Hyp^3^,Thi^5^,D-Tic^7^,Oic^8^]BK) can be considered marker bands for peptides deposited on the AuNP sensors.

## Figures and Tables

**Table 1:** Primary structure of highly selective bradykinin B2 receptor antagonists.

Peptide	Peptide Sequence									
	0	1	2	3	4	5	6	7	8	9
Native BK	–	Arg	Pro	Pro	Gly	Phe	Ser	Pro	Phe	Arg
[D-Arg^0^,Hyp^3^,Thi^5^,D-Phe^7^,Thi^8^]BK	D-Arg	Arg	Pro	**Hyp**	Gly	**Thi**	Ser	**D-Phe**	**Thi**	Arg
[D-Arg^0^,Hyp^3^,Thi^5^,D-Tic^7^,Oic^8^]BK	D-Arg	Arg	Pro	**Hyp**	Gly	**Thi**	Ser	**D-Tic**	**Oic**	Arg
[D-Arg^0^,Hyp^3^,Igl^5^,D-Phe(5F)^7^,Oic^8^]BK	D-Arg	Arg	Pro	**Hyp**	Gly	**L-Igl**	Ser	**D-Phe(5F)**	**Oic**	Arg
[D-Arg^0^,Hyp^3^,Igl^5^,D-Igl^7^,Oic^8^]BK	D-Arg	Arg	Pro	**Hyp**	Gly	**L-Igl**	Ser	**D-Igl**	**Oic**	Arg
Hyp L-hydroxyproline					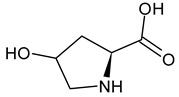					
Thi L-thienylalanine					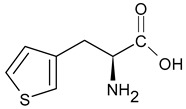					
D-Tic D-1,2,3,4-tetrahydroisoquinoline-3-carboxylic acid					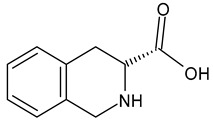					
Igl 2-indanylglycine					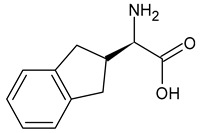					
Oic L-octahydroindole-2-carboxylic acid					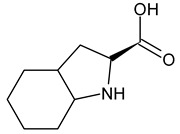					

**Table 2:** Proposed assignment of observed bands in Raman and SERS spectra.

Assignment	Wavenumber/cm^−1^											
	[D-Arg^0^,Hyp^3^, Thi^5^, D-Phe^7^,Thi^8^]BK			[D-Arg^0^,Hyp^3^,Thi^5^, D-Tic^7^,Oic^8^]BK			[D-Arg^0^,Hyp^3^,Igl^5^,D-Phe(5F)^7^,Oic^8^]BK			[D-Arg^0^,Hyp^3^,Igl^5^, D-Igl^7^,Oic^8^]BK		
	Raman	SERS Ag	SERS Au	Raman	SERS Ag	SERS Au	Raman	SERS Ag	SERS Au	Raman	SERS Ag	SERS Au
Phe(F5) [ν_8a_]							1682					
Amide I/Arg ν_as_(C=N)	1660	1565	1546	1663	1586	1522	1659	1585	1591	1676	1586	1564
Phenyl ring [ν_8a_]	1603		1592	1610		1583	1607		1560	1606		1598
Phenyl ring [ν_8a_]	1584			1590			1584			1585		
ν_as_(COO^−^)	1534		1520	1533		1522			1509			1527
δ(CH_2_) + Imide II/Arg ρ_asb_(CH_2_)/a-ring ρ_s_(CH_2_)	1458	1456	1449	1461	1457	1490	1464	1455		1468	1456	1485
δ(CH_2_) and Imide II	1437			1438	1438		1445		1442	1446	1437	1444
ν_s_(COO^−^)			1381		1397	1393					1397	1404
Arg ρ_b_(CH_2_)/Thi δ(CH) + ν(C=C) and a-ring combination	1366	1352		1343	1352	1364	1343	1352	1343	1343	1352	1367
a-ring ν(CN), δ(CC_α_H)			1305	1322		1322	1315			1323		
a-ring [ν(CN) + ρ_b_(CCH) + ρ_r/t_(CH_2_)]/Imide II	1294	1284	1276		1296			1296	1294		1290	
Thi ρ_r_(CH)/a-ring ρ_t_(CH_2_)				1262		1283	1263			1261		1272
Amide III	1248		1244	1246			1247		1239	1248		1237
Phenyl ring [ν_7a_]	1201		1223	1201			1208		1198	1209		1215
Arg ρ_r_(NH_2_)/a-ring ρ_t_(CH_2_)	1167	1166	1180	1158		1174	1154	1166		1153	1139	1169
a-ring ρ_t_(CH_2_)	1127				1123			1123	1134		1123	1127
ν(CN)	1097			1096	1087	1080	1088			1088	1087	
Arg ν(CC) + ν(NC)/a-ring ν(CC)	1061			1074			1066			1066		
Igl ν(CC) + ν(NC)							1045			1044		
Thi ν(CC) + ρ_r_(CH)/Arg guanidino group/δ(a-ring) + ρ_t_(CH_2_)/Phe [ν_18a_]			1013	1034		1034	1024		1024	1024		1020
Phe [ν_12_]	1001		996									
Igl							1011			1011		
ρ_r_(CH_2_)			982	990		959	987		988	987		991
Arg ν(CC)/a-ring ρ_r_(CH) /ρ_w_(CH_2_) + γ(ring)/[ν(CC_OO–_)],			906	925	925		924			928	925	912
Thi δ(ring) + ν(CS)/a-ring ν(CCC)	888		880			883	870			870		
Thi δ(ring) + ν(CC)/Arg ν(CC)/a-ring (ρ_r_(C_β_H_2_)	850		836	851		833			824			
Thi δ(ring) + ν(CC)/a-ring breath		811					817	811		817		829
a-ring (ρ_r_(C_γ_H_2_)						784	789			788		
Thi δ(ring) + ν(CS)/a-ring ρ_r_(CH_2_) + ρ_b_(CNH)	741		732	742		742						
Thi ring ρ_w_(CH)	673	673	653	662	674	663		675	664		673	666
ρ_b_(COO)/Amide V	649		648	599	653	648	607			585		606
Arg ρ_t_(NH_2_)/a-ring δ(CCN)/Phe(F5)	505		501	506		551	564			542		
γ(ring)	465					481	445		446			461

Abbreviations: ν—stretching, δ—deformation, ρ_r_—rocking, ρ_w_—wagging, ρ_b_—bending, ρ_t_—twisting, ρ_s_—scissoring, γ—torsional, s—symmetric, as—asymmetric vibrations, Arg—L-arginine, Phe—phenyl ring, and Thi—thienyalalanine.

**Table 3:** Proposed assignment of observed bands in ATR-FTIR and SEIRA spectra.

Assignment	Wavenumber/cm^−1^											
	[D-Arg^0^,Hyp^3^,Thi^5^,D-Phe^7^,Thi^8^]BK			[D-Arg^0^,Hyp^3^,Thi^5^,D-Tic^7^,Oic^8^]BK			[D-Arg^0^,Hyp^3^,Igl^5^,D-Phe(5F)^7^,Oic^8^]BK			[D-Arg^0^,Hyp^3^,Igl^5^,D-Igl^7^,Oic^8^]BK		
	ATR-FTIR	SEIRA Ag	SEIRA Au	ATR-FTIR	SEIRA Ag	SEIRA Au	ATR-FTIR	SEIRA Ag	SEIRA Au	ATR-FTIR	SEIRA Ag	SEIRA Au
ν(C=O)	1729			1729		1750	1728		1750	1724		1735
Amide I [ν(C=O)]/Arg ν_as_(C=N), δ(NH_2_)	1647	1660	1660 1640	1632	1665	1631	1638	1664	1658 1626	1648	1653	1659 1620
ν_as_(COO^−^)		1580			1579			1580			1572	
Amide II [ν(NC) + ρ_ipb_(NH)]	1537	1533	1533	1545 1530		1548 1533	1545		1551	1548	1548	1555
Imide II/Arg ρ_as b_(CH_2_)	1470			1467		1456	1470		1457	1471		1465
a-ring ρ_s_(CH_2_)	1456	1433	1457	1447	1447		1450	1450		1452		1452
ν_s_(COO^−^)		1397			1396	1396		1398	1396		1393	
ρ(CH_2_)		1368		1339	1367		1339	1365			1364	
Amide III	1251	1251	1251	1251	1251	1252	1251	1261	1251	1251	1259	1251
ρ_r_(CH_2_), ν(CC)				1202		1197	1196		1197	1203		
ν(CN)	1068	1078	1050	1068	1074	1067	1068	1078	1058	1068	1078	1080
ρ_r_(CH_2_)				976			977					956
Thi δ(ring) + ν(CC)/Arg ν(CC), δ(NH)/a-ring ν(CC)	842	841		843			842	840		842	842	
δ(NH)	788			757			788			789		
Skeletal	719			709			718			721		
Amide	651	638			638		684	639		679	637	

Abbreviations: ν—stretching, δ—deformation, ρ_r_—rocking, ρ_w_—wagging, ρ_b_—bending, ρ_t_—twisting, ρ_s_—scissoring, γ—torsional, s—symmetric, as—asymmetric vibrations, Arg—L-arginine, Phe—phenyl ring, and Thi—thienyalalanine.

## Data Availability

Data are available upon request (proniewi@agh.edu.pl).

## Abbreviations

The following abbreviations are used in this manuscript:

ATR-FTIR	Attenuated total reflection Fourier transform infrared spectroscopy
AuNPs	Gold nanoparticles
BK	Bradykinin
FWHM	Full width at half maximum
GPCR	G-protein-coupled superfamily
Hyp	L-hydroxyproline
Igl	2-indanylglycine
Oic	L-octahydroindole-2-carboxylic acid
Phe(5F)	D-pentafluorophenylalanine
SEIRA	Surface-enhanced infrared spectroscopy
SERS	Surface-enhanced Raman scattering
SEM	Scanning electron microscopy
D-Tic	D-1,2,3,4-tetrahydroisoquinoline-3-carboxylic acid
Thi	L-thienylalanine
TERS	Tip-enhanced Raman scattering
AgNPs	Silver nanoparticles
SCLC	Small cell lung cancer
UV-Vis	Excitation spectroscopy
